# Case report of small bowel obstruction caused by small intestinal metastasis of bilateral breast cancer

**DOI:** 10.3892/etm.2013.1220

**Published:** 2013-07-12

**Authors:** LIQIONG LV, YUN ZHAO, HUI LIU, ZHONGYI PENG

**Affiliations:** 1 Department of Liver and Gall Surgery, Ruikang Hospital Affiliated to Guangxi University of Chinese Medicine, Nanning, Guangxi 530001, P.R. China; 2 Department of Gastrointestinal Surgery, Ruikang Hospital Affiliated to Guangxi University of Chinese Medicine, Nanning, Guangxi 530001, P.R. China; 3 Department of Pathology, Ruikang Hospital Affiliated to Guangxi University of Chinese Medicine, Nanning, Guangxi 530001, P.R. China

**Keywords:** bilateral breast cancer, small intestine metastatic breast cancer

## Abstract

A 41-year-old female was admitted into hospital due to recurrent abdominal pain with bloating. An enteroscopy was carried out and stenosis in the lower jejunal lumen was identified. This led to a diagnosis of small bowel obstruction caused by inflammation. During the laparotomy, the resection and anastomosis of a narrow segment of small intestine was performed. In combination with the results of immunohistochemical analysis, the postoperative pathology indicated the presence of a poorly differentiated/undifferentiated carcinoma of the small intestine, which was considered to have arisen from breast cancer. Postoperative examination showed bilateral breast masses, and the pathology of the right breast tumor biopsy prompted the diagnosis of invasive lobular carcinoma. A breast MRI was reviewed following five cycles of XT chemotherapy and the evaluation was stable disease (SD). Since the mass was not sensitive to chemotherapy, a bilateral modified radical mastectomy was performed, and postoperative pathology confirmed the mass to be primary bilateral invasive lobular carcinoma.

## Introduction

Bilateral primary breast cancer (BPBC), a type of primary breast cancer, has an incidence rate of between 2 and 11% (1), of which bilateral invasive lobular carcinoma accounts for 6–28%. Invasive lobular carcinoma exhibits a specific pathology and the likelihood of developing synchronal or metachronous bilateral breast cancer is two-fold that of invasive ductal carcinoma. Common sites of invasive ductal carcinoma metastasis are the liver, lungs and bones, whereas invasive lobular carcinoma more readily metastasizes to the peritoneum, retroperitoneum and the genitourinary system, in addition to the gastrointestinal tract (2). In this case report, we describe a case of small bowel obstruction in our hospital, which was diagnosed as metastatic adenocarcinoma of the small intestine caused by primary invasive lobular breast cancer.

## Case report

**Figure 1:**
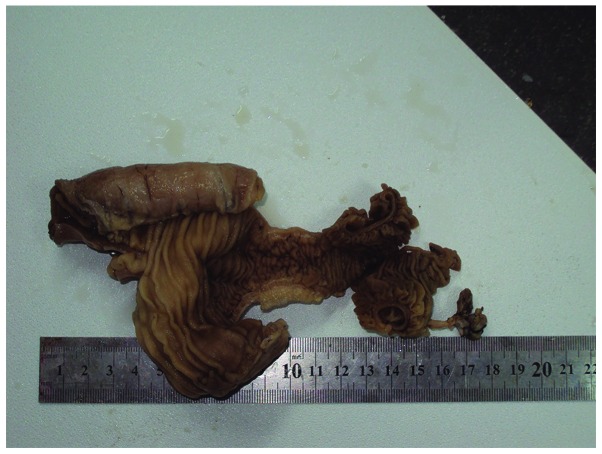
Sample of the narrow small bowel resection.

**Figure 2:**
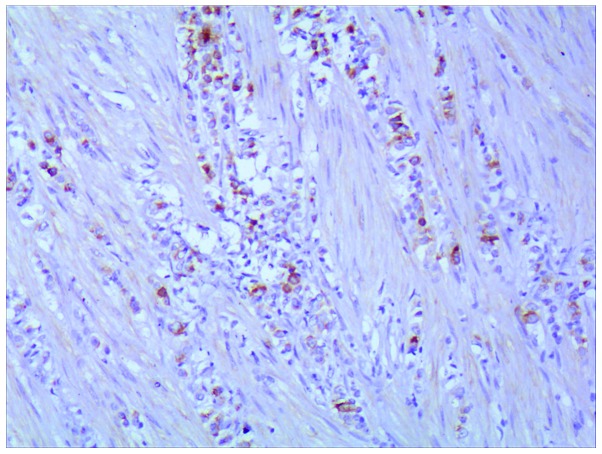
Metastatic poorly differentiated adenocarcinoma of the small intestine: Gross cystic disease fluid protein 15 (GCDFP)-15 (+) (amplified 20×10).

**Figure 3:**
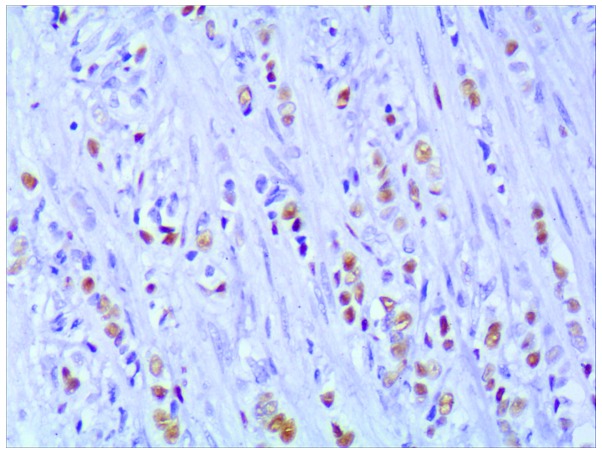
Metastatic poorly differentiated adenocarcinoma of the small intestine: estrogen receptor (ER) (+) (amplified 40×10).

A 41-year-old female was admitted to our hospital (Ruikang Hospital Affiliated to Guangxi University of Chinese Medicine, Nanning, China), presenting with recurrent abdominal pain (apparent for >1 year) and paroxysmal abdominal pain accompanied by bloating and loose stools, but no nausea or vomiting. The results of the physical examination revealed abdominal distension and visible intestinal peristaltic waves; however, there was no whole abdominal or rebound tenderness. Orthostatic images of the abdomen showed an incomplete intestinal obstruction, while the results of an oral colonoscopy revealed a narrow lower end of the jejunum, no ulceration and a new neoplasm, which was considered to be narrow small intestine (possibly the lower end of the jejunum). The narrow section was surgically biopsied (2011–6099) and judged to be a chronic mucosal inflammation of the lower end of the jejunum. Laparotomy revealed a hardening and narrowing of the small bowel at the junction of the jejunum and ileum, with significant expansion proximal to the jejunum and a large volume of intestinal contents. Numerous lymph nodes were observed on the mesentery, and the intestinal canal had significant hyperemia and edema. There was a section of diseased small intestine ~1.5 m to the ileocecal valve, and the lumen was hardened and narrow. The two mucous membranes of the small intestine exhibited pathological changes and were smooth and complete. The lesions did not penetrate the mucous layer. Small bowel resection and anastomosis surgery were performed on the narrow section (Fig. 1). The postoperative pathological results revealed a poorly differentiated/undifferentiated carcinoma of the small intestine, with invasion of the cancerous tissue into the intestinal submucosa, muscularis externa and placenta percreta. Extensive invasion of the cancerous tissue was also observed inside the mesenteric layer; however, there was no cancer present in the mucous layer and no metastasis into the intestinal lymph node (0/2; number of lymph nodes with metastasis/the total number of intestinal lymph nodes). Immunohistochemical analysis showed that the tumor cells were positive for cytokeratin (CK) (+), epithelial membrane antigen (EMA) (+), cell proliferation nuclear antigen Ki-67 (+5–10%), gross cystic disease fluid protein 15 (GCDFP-15) (+) (Fig. 2), estrogen receptor (ER) (+) (Fig. 3) and progesterone receptor (PR) (+) ; other indicators, such as vimentin (Vim), synaptophysin (Syn), chromogranin A (CgA), Acid calcium binding protein S-100, smooth muscle actin (SMA), leucocyte common antigen (LCA) and carcinoembryonic antigen (CEA), were negative. Combined with the immunohistochemistry results, it was recommended that the primary tumor from the lacteal gland be located. A postoperative examination showed that both breasts were symmetrical, while there was a goiter measuring ~3×3 cm in the upper outer quadrant of the right breast, and another goiter measuring ~2×1.5 cm just above it. The two goiters were hard, without clear borders. In addition, a 4×3 cm hard goiter was felt at the right armpit. The color Doppler results revealed a hypoechoic mass of ~15×29×7 mm in the 10 o’clock position of the right breast, a hypoechoic mass of ~14×8 mm in the 12 o’clock position of the right breast and a hypoechoic mass of ~15×7 mm in the 12 o’clock position of the left breast. Punctiform blood flow signals were observed inside and at the edge above the hypoechoic mass. A hypoechoic nodule, measuring ~8×5 mm, was probed in the right armpit, while no swelling of the lymph nodes was detected in the left armpit. With regard to the thyroid, the bilateral lobes were positively substantiality mass, and the internal right lobe was fluidified and calcified. The pathology results for the biopsy of the right breast goiter revealed that the goiter was an infiltrating lobular carcinoma; lobular carcinoma *in situ* was visible peripherally (accounting for 80%). Tests showed ER 80%, PR 70%, CerbB-2 (−) and Ki-67 (−). The pathology results for the biopsy of the thyroid indicated a thyroid follicular adenoma.

**Figure 4:**
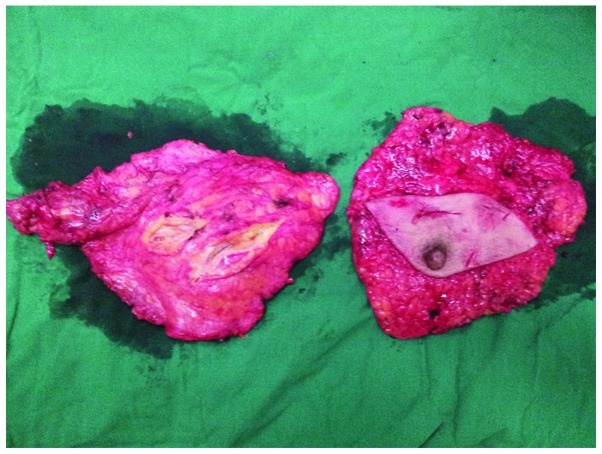
Sample of the bilateral modified radical mastectomy.

**Figure 5:**
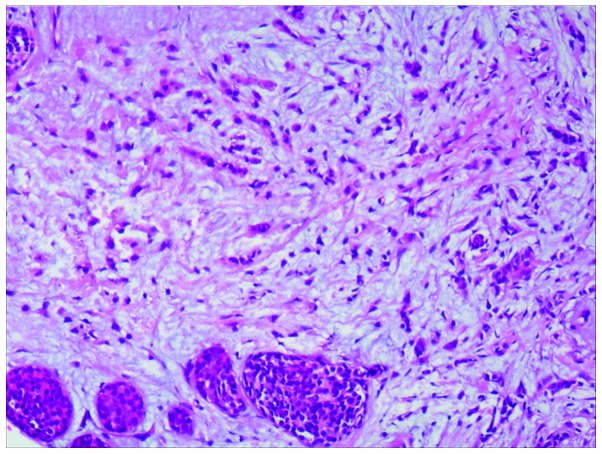
Infiltrating lobular carcinoma of the right breast and lobular carcinoma *in situ* (amplified 20×10).

**Figure 6:**
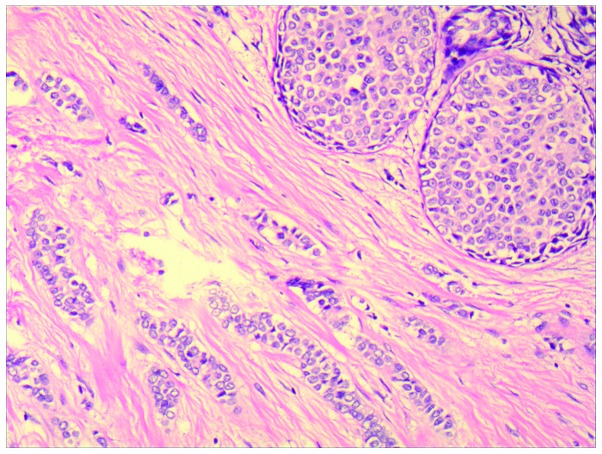
Infiltrating lobular carcinoma of the left breast and lobular carcinoma *in situ* (amplified 20×10).

Following two cycles of XT chemotherapy (paclitaxel, 210 mg, 175 mg/m^2^ and Xeloda, 2,500 mg, 1,250 mg/m^2^), MRI results indicated that the goiter on the upper outer quadrant of right breast was patchy, measuring ~17×35×34 mm, with inhomogeneous enhancement, and the time-signal intensity curve was of the rising type. MRI examination following five cycles of chemotherapy revealed that the goiter on the upper outer quadrant of the right breast was irregular, with a trend of fusion, ~17×35 cm and 17×16 cm. The time-signal intensity curve was of the rising type. According to the Response Evaluation Criteria in Solid Tumors (RECIST), the evaluation was stable disease (SD). A bilateral modified radical mastectomy was performed (Fig. 4), and the pathology results demonstrated that the right breast had an invasive lobular carcinoma (Fig. 5) with two lesions (~3×2×2 cm and 5×4×3 cm, respectively), axillary lymph node 29/31 metastasis, ER 8%, PR 90%, CerbB-2 (+/−) and Ki-67 <3%. The left breast had an invasive lobular carcinoma (Fig. 6) with two lesions, there was no metastasis in all 17 axillary lymph nodes, ER (−), PR 50~75%, CerbB-2 (−) and Ki-67<5%. It was therefore recommended that the patient undergo an oophorectomy and commence endocrine therapy.

## Discussion

Bilateral primary breast cancer (BPBC), a type of primary breast cancer, has an incidence rate of between 2 and 11% (1) and between 1.4 and 7.7% in China (3). The pathology includes invasive lobular carcinoma (4.8%) (4). A retrospective analysis from McLemore *et al*(5) revealed that 1,516 out of 12,550 cases of breast cancer in a 15-year period were invasive lobular carcinoma, accounting for 12%. Invasive lobular carcinoma has specific cancer characteristics and is relatively rare, compared with other types of invasive breast cancer. Invasive lobular carcinoma has an ipsilateral multifocal breast characteristic, and bilateral cancers are frequently observed (6). It has negative characteristics of PR+, ER+, HER-2, P53 and EGFR (7). In the current case, the breast tumor biopsy results prior to chemotherapy were PR+, ER+ and HER-2 (−), which were consistent with the literature. The postoperative pathology indicated ER (−), and the lobular carcinoma *in situ* prior to chemotherapy accounted for 80%. Following the chemotherapy, there was no obvious *in situ* carcinoma component. These changes were considered to result from the impact of chemotherapy on the breast cancer cells. It has been suggested that gastrointestinal metastasis occurs more commonly in breast invasive lobular carcinoma and in the PR+, rather than in the non-ER+ subtype (8).

The cells of invasive lobular carcinoma are small with poor tumor cell adhesion and cohesion. However, they demonstrate strong invasive power and may easily break into the vasculature, penetrate the basement membrane and enter the lymph or the blood circulation. Therefore, lymphatic and distant metastases of invasive lobular carcinoma are more common, resulting in poor prognosis. In the review by McLemore e*t al*(5), out of 12,001 cases of metastatic breast cancer, only 23 cases had gastrointestinal metastasis alone. Primary invasive lobular carcinoma accounted for 54% of these cases, a significantly higher proportion than the other pathological types (P<0.001). In addition, there were two cases (3%) of bilateral breast cancer and six cases (8%) where contralateral secondary breast cancer occurred prior to metastasis. In 45% of cases there was metastasis to the colon and rectum, while metastasis to the stomach and small intestine occurred in 28 and 19% of cases, respectively. Borst and Ingold analyzed the pathology of more than 2,500 cases of metastatic breast cancer in 18 years, and revealed that gastrointestinal metastasis was apparent in only 17 cases (<1%) (9). Therefore, this suggests that gastrointestinal metastasis from breast cancer is rare.

It has been demonstrated that invasive lobular carcinoma and invasive ductal carcinoma exhibit different metastatic characteristics, with lobular carcinoma metastasizing more rarely than ductal carcinoma, particularly to the lungs, liver and brain parenchyma (2). Instead, lobular carcinoma has the tendency to metastasize to the pia mater, peritoneal surface, retroperitoneum and the gastrointestinal and reproductive organs. Cifuentes and Pickren (10) identified 112 (16%) cases with gastrointestinal metastasis of primary breast cancer in 707 autopsies (10), among which the small intestine accounted for 64 cases (9%), the stomach for 69 cases (10%) and the large intestine for 57 cases (8%). Therefore, this study suggests that gastrointestinal metastasis of breast cancer is not uncommon. The clinical manifestations of the majority of gastrointestinal diseases include abdominal pain, diarrhea, gastrointestinal bleeding, intestinal obstruction and intussusception. Since these manifestations are also apparent in patients with gastrointestinal metastasis, there may be problems diagnosing gastrointestinal metastasis, which may lead to treatment difficulties (11). The patient admitted into hospital in the present case exhibited symptoms of acute intestinal obstruction and the doctors and patient ignored the breast disease; therefore, breast disease was not diagnosed preoperatively. Postoperative pathology prompted the discovery that the primary disease responsible for the small intestine metastasis was in the breast. We concluded that there were two main errors in the case. One factor was a lack of awareness of the disease in the patient: The patient had known for several years that breast and thyroid masses were present; however, since they did not affect the patient’s life, they were not considered to be a disease and the patient did not report them to a doctor. The gastrointestinal obstructive symptoms severely impacted the patient’s life, and it was only this situation that caused the patient to go to the hospital. Another factor was that the doctor did not demonstrate sufficient understanding: When the preoperative colonoscopy revealed no evidence of a tumor, the bowel obstruction was considered to be mere inflammation. Therefore a thorough and comprehensive medical history and physical examination was necessary.

Following the small intestine tumor resection, and according to the pathology of the breast tumor puncture, the patient was diagnosed with advanced breast cancer with symptomatic visceral metastasis. Subsequent to the surgery, chemotherapy was initially considered to control the systemic metastasis, and the patient was treated with paclitaxel in combination with capecitabine (paclitaxel, 175 mg/m^2^ day 1, capecitabine, 1,250 mg/m^2^ days 1–14, 21 days for one cycle). Following five cycles of chemotherapy, the breast mass of the patient was evaluated to be SD, leading to a bilateral modified radical mastectomy being proposed. The failure of chemotherapy was due to the breast mass being lumimal A type, and, therefore, not sensitive to chemotherapy, while sensitive to hormone therapy. Seewaldt *et al*(12) described four cases where intestinal perforation occurred when small intestinal metastatic tumors were treated with paclitaxel chemotherapy (12). The perforation was revealed not to be caused by the dissolution of the tumor, since three cases progressed. McLemore *et al*(5) concluded that the overall survival following gastrointestinal metastasis of breast cancer was 28 months (5), with age and diagnosis of gastrointestinal metastasis having no effect on overall survival; however, systematic chemotherapy and tamoxifen treatment were considered to be influential factors. The patient in the present case underwent an oophorectomy and was treated with letrozole for endocrine therapy following the bilateral modified radical mastectomy. It was indicated that the endocrine therapy was more efficacious than the chemotherapy.

The clinical symptoms and signs of small intestine metastatic tumors have no identifiable features when compared with other diseases of the small intestine, leading to certain difficulties in the diagnosis. Gastrointestinal tract metastasis may be apparent in cases of invasive lobular carcinoma; however, intestinal metastasis in cases of bilateral breast infiltrating lobular carcinoma is clinically rare. As a consequence, doctors and patients have a tendency to ignore the signs and symptoms of intestinal metastasis of invasive lobular cancer. This case reinforced the fact that it is necessary to consider the possibility of metastasis when diagnosing gastrointestinal diseases, and that there is potential for gastrointestinal tract metastasis in cases of invasive breast lobular carcinoma.
